# Supplementary material for: Lysyl oxidase-like 1 predicts the prognosis of patients with primary glioblastoma and promotes tumor invasion via EMT pathway
Source: PeerJ. 2024 Jul 5;12:e17579. doi: 10.7717/peerj.17579 (PMC11229686; doi:10.7717/peerj.17579)
Supplement: Supplemental Information 7 [file peerj-12-17579-s007.docx]

**Contents：**

**1. Supplemental Figure 1**

**
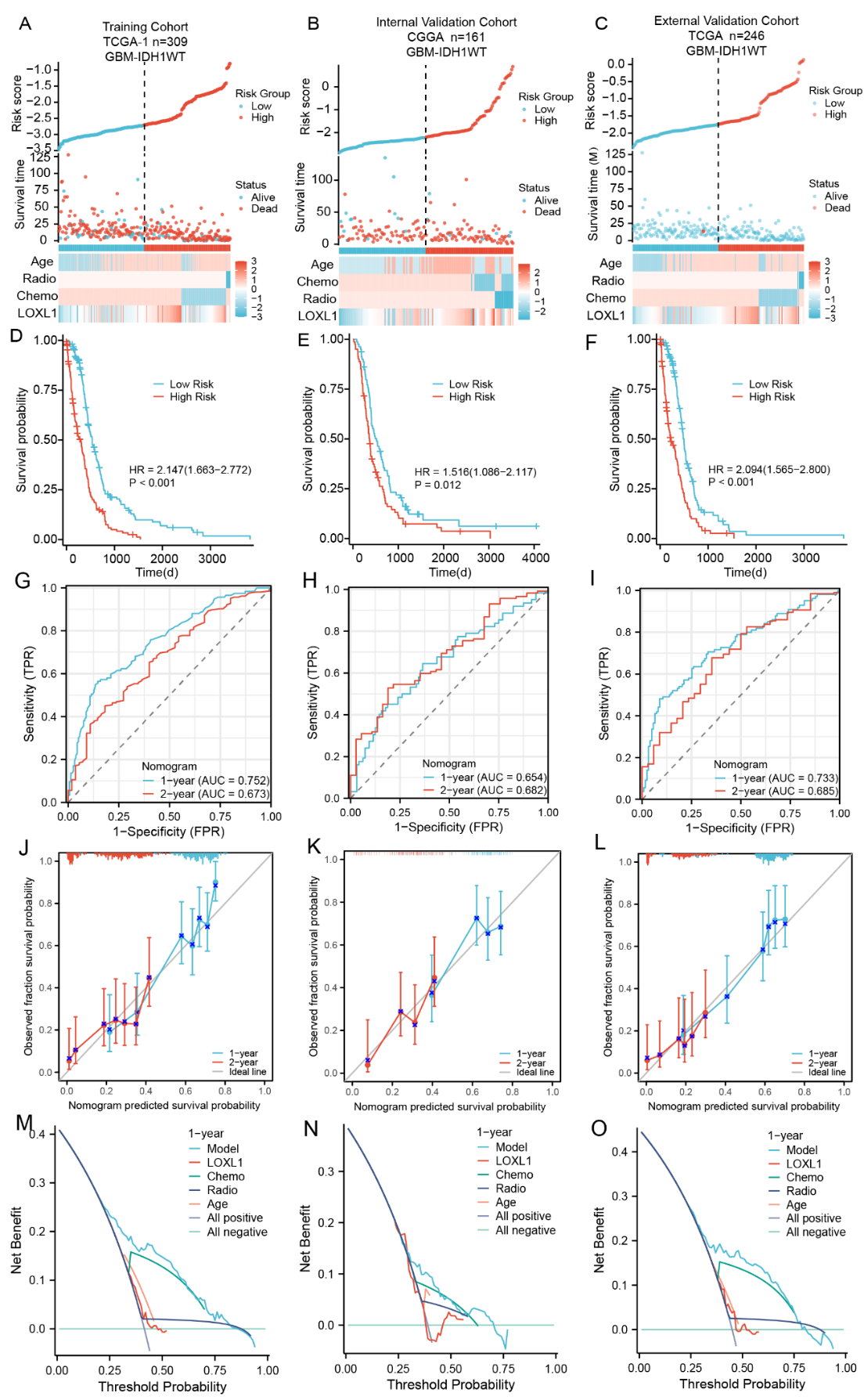
**

**Supplemental Fig****ure 1. Distribution and evaluation of Loxl1-based nomogram model in IDH1-WT GBM cohorts** Risk scores, clinicopathological factors, and survival time in IDH1-WT GBM patients were exhibited in the dots plot and heatmaps (the training TCGA (**A**), CGGA (**B**), and external TCGA (**C**) cohorts). Kaplan-Meier analyses were also conducted (the training TCGA (**D**), CGGA (**E**), and external TCGA (**F**) cohorts). The AUC exhibited the performance that the model predicted 1- and 2-year survival time (the training TCGA (**G**), CGGA (**H**), and external TCGA (**I**) sets). The 1-year prediction was in blue, and the 2-year was in red. The calibration plots compared the model prediction and actual observations. The 1-year predictions were represented in blue, while the 2-year predictions were delineated in red across distinct sets, including the training set (**J**), CGGA dataset (**K**), and the external TCGA dataset (**L**). The DCA presented the different net benefits from the model and clinical features (the training (**M**), CGGA (**N**), and external TCGA (**O**) sets).

**2. Supplemental Table 1-5**

**Supplemental Table 1.** Complete mutation and copy number alteration (CNA) data from three GBM cohorts

**Supplemental Table 2.** The top 50*5 genes most related to LOXs in GBM

**Supplemental Table 3.** The training cohort from the TCGA GBM database.

**Supplemental Table 4.** The internal cohort from the CGGA GBM database.

**Supplemental Table 5.** The external cohort from another TCGA GBM database.

All above files were attached in supplementary materials.

**3. Supplemental Table 6** Marker genes for cell-type annotation of GSE182109 in single cell RNA sequencing analysis.

| **Cells** | **Feature genes** | **Clusters** |
| --- | --- | --- |
| Myeloid cells | PTPRC/CD45, ITGAM/CD11B, CD68 | C0, C3 |
| Glioma cells | SOX2, OLIG1, GFAP, S100B | C1, C2, C5, C7, C8, C10, C11 |
| T cells | PTPRC/CD45, CD3E, CD4, CD8A | C4 |
| Pericytes | ACTA2, PDGFRB | C9 |
| Endothelial cells | PECAM | C12 |
| Oligodendrocytes | OLIG2, MBP | C6 |
