# Supplementary material for: Lysyl oxidase-like 1 predicts the prognosis of patients with primary glioblastoma and promotes tumor invasion via EMT pathway
Source: PeerJ. 2024 Jul 5;12:e17579. doi: 10.7717/peerj.17579 (PMC11229686; doi:10.7717/peerj.17579)

Original blots and images:

Original blots (**A**) Western blots of knockdown-Loxl1. N = 3. (**B**) Western blots of N-cadherin and (**C**) E-cadherin under the intervention of Loxl1. N = 3. **(D)** Western blots of Vimentin and **(E)** Snail under the intervention of Loxl1. N = 3. The specific bands were pointed by arrows.

A. Original blots of Figure 7A

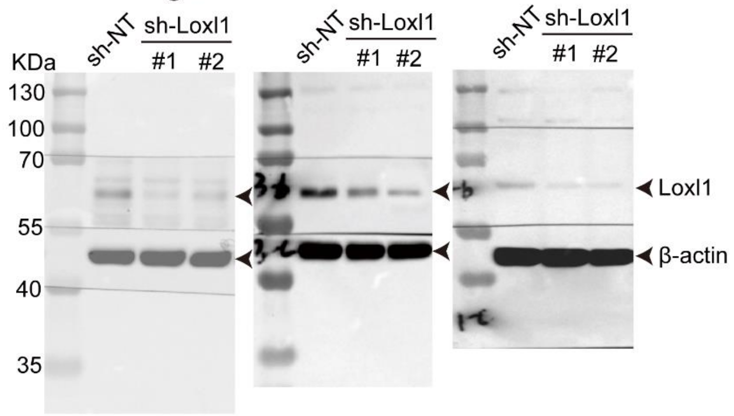

B. Original blots of Figure 7C

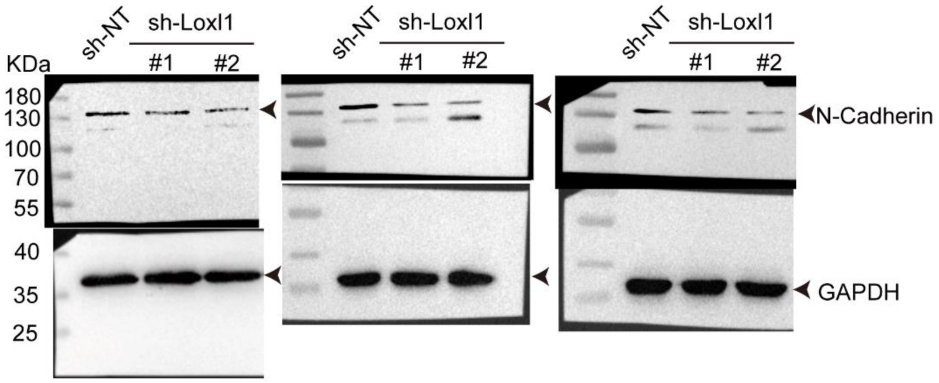

C. Original blots of Figure 7E

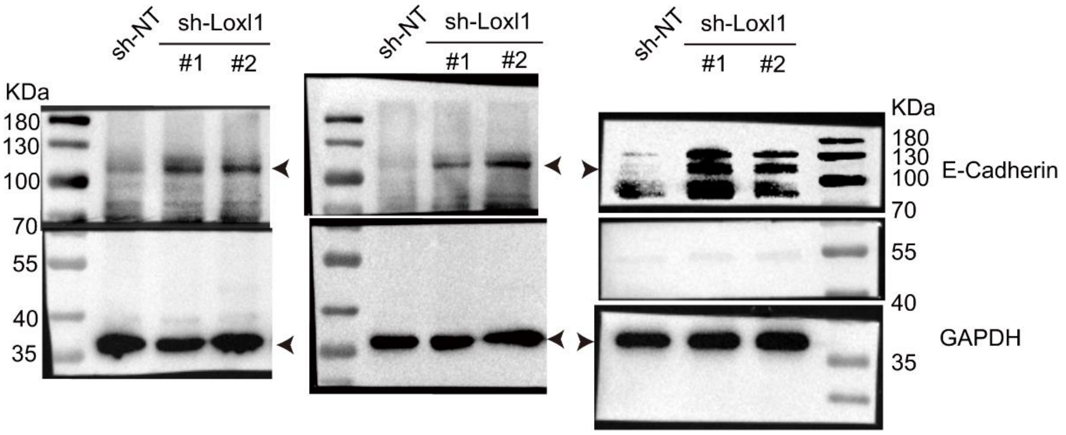

D. Original blots of Figure 7H

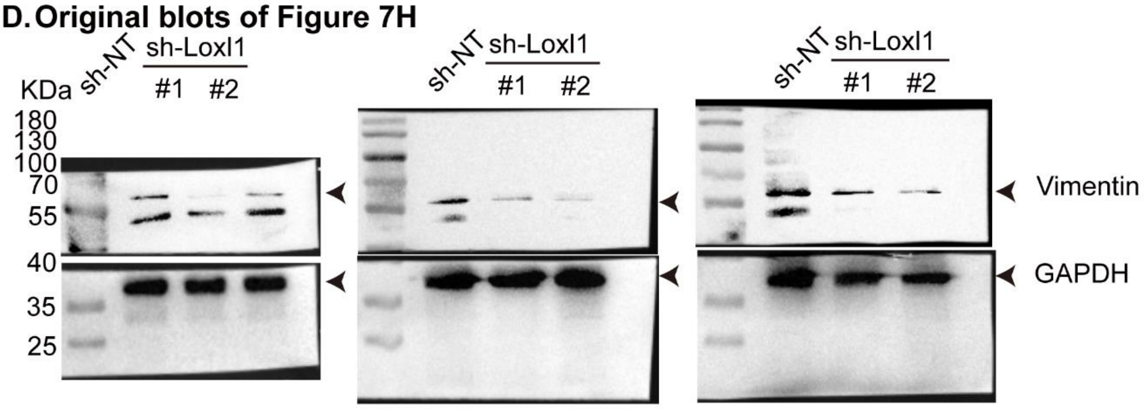

E. Original blots of Figure 7J

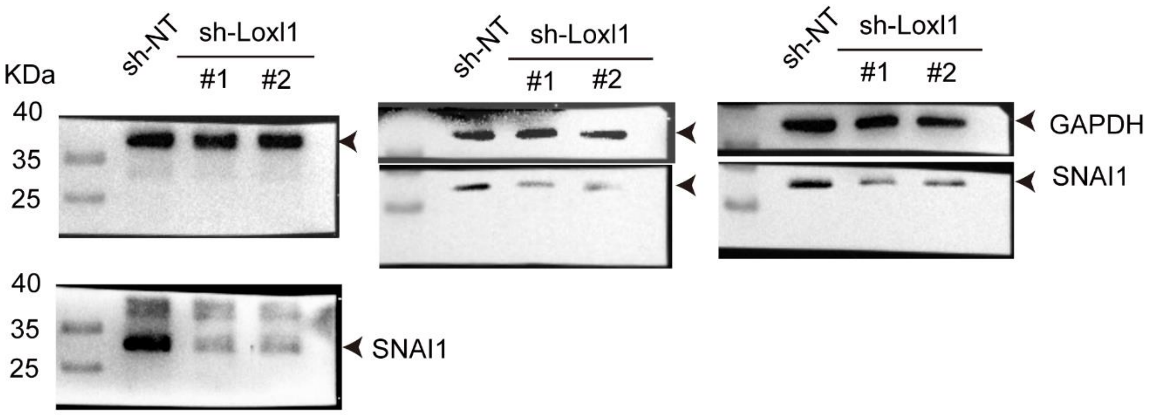

Original images (**A**) The wound healing assay of Loxl1-knockdown glioma cells compared to the control. The gap was measured at 0 h and 24 h after the scratch. The black scale bar was 200 μm. N = 3. (**B**) Transwell invasion assay of Loxl1-knockdown glioma cells. The white scale bar represented 200 μm. N = 3.

A.Original images of Figure 7G

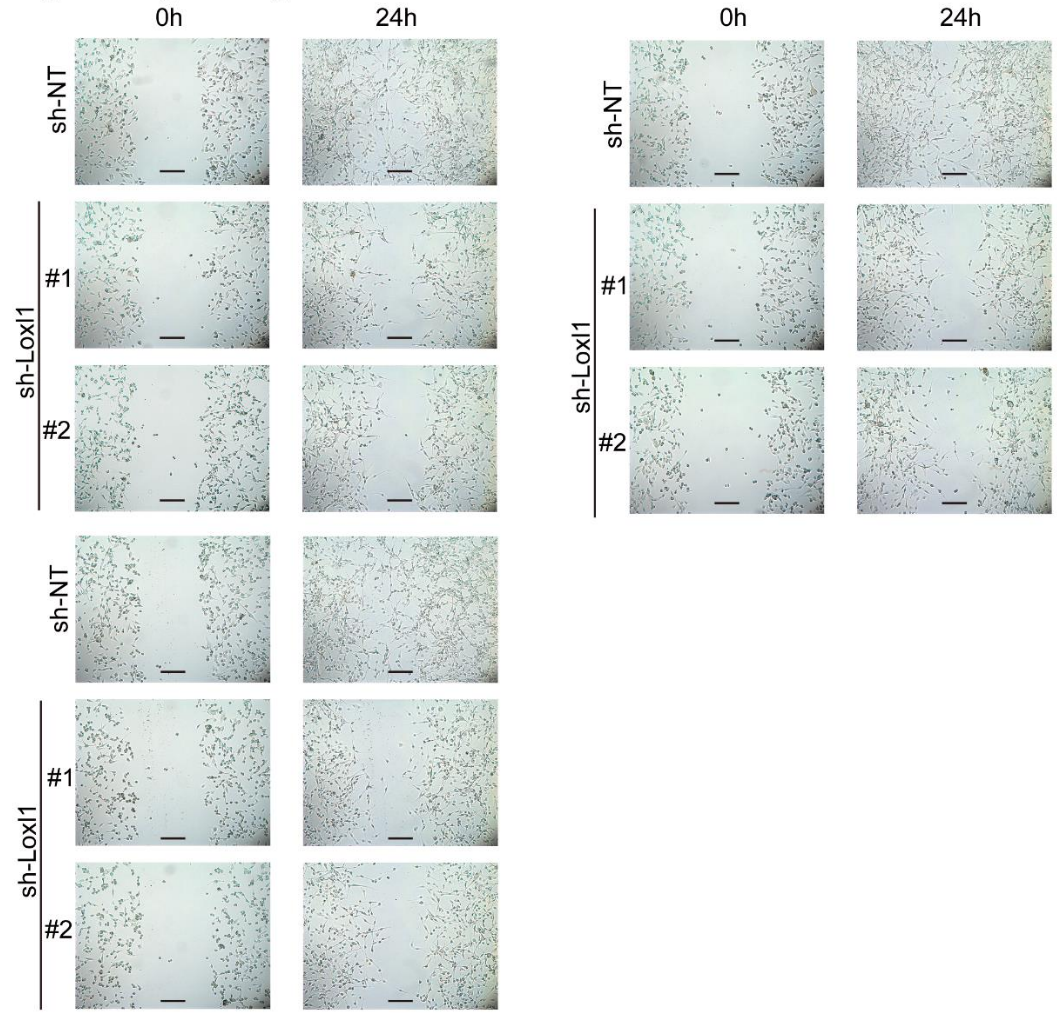

B.Original images of Figure 7I

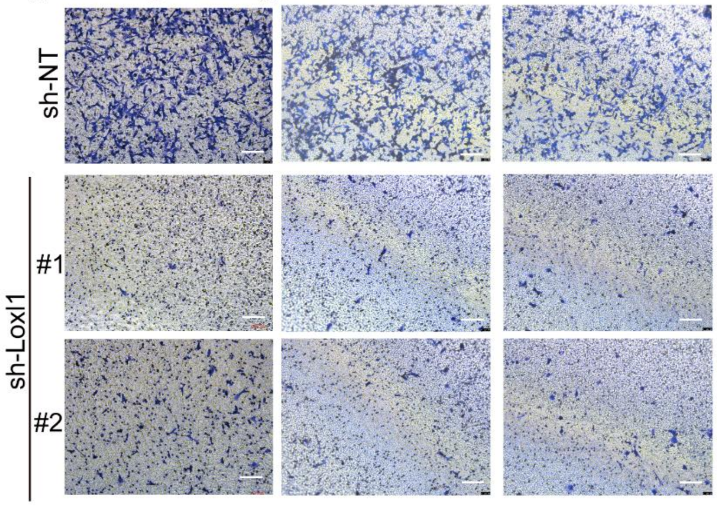

Supplement: Supplemental Information 8 [file peerj-12-17579-s008.pdf]
